# Does Reading Words Differing in Arousal Load Influence Interference Control in Flanker Task?

**DOI:** 10.1007/s12144-015-9396-9

**Published:** 2015-12-11

**Authors:** Kamil K. Imbir

**Affiliations:** Faculty of Psychology, University of Warsaw, ul. Stawki 5/7, 00-183 Warsaw, Poland

**Keywords:** Semantic priming, Words reading, Arousal effect, Flanker competition

## Abstract

Arousal involves a physiological and psychological state of being awake or reactive to stimuli. It could be treated also as an energetic property of stimulation. On the basis of previous findings concerning affective state modulation of spatial processing, I predict that arousal impact will follow the Yerkes-Dodson law. To test this hypothesis, 135 words were chosen and divided into three levels of arousal (low, medium and high), whilst controlling for valence, concreteness, frequency of appearance and length. Forty-nine individuals performed a flanker task while reading the words in order to provide a measure of interference control over spatial processing. The accuracy of answers, reaction times and interference effect index were analyzed. It appears that, at the medium arousal level of words, arousal was optimal for interference control, while both low and high arousal impaired the cognitive control of interference caused by competing flanker and target stimuli features.

## Introduction

Much of our visual cognition concerns space and position in relation to other objects (c.f., Lavie et al. 2004; Lavie and Dalton 2014). Visual objects share many features that compete to create a perceptual representation of a word in our minds. The interference between target and non-target object features was found to be responsible for some difficulties in visual processing (Eriksen and Eriksen 1974). This is especially so when people are expected to react to specific types of objects, and inhibit their reactions to other, similar types of objects presented at the same time; interference processing costs then occur. This means that participants react slower or less accurately to visual stimuli that produce an interference in comparison to non-interfering objects.

The costs observed are thought to be manifestations of cognitive control, namely interference control (c.f., Nee and Jonides 2008). defined as the ability to inhibit inaccurate cognitions or responses. In other words, this concept is relevant to both working memory function (cognitive inhibition) and motor control (response inhibition: Nigg 2000). The degree of cognitive control exerted on a particular task appears to reflect not only the quality, but also the quantity of recent experiences of information-processing conflicts (Freitas et al. 2007). It has been noted that interference control is sensitive to the affective states of individuals in the way that affect shapes the effectiveness of maintaining control over unwilling or unrelated-to-the-task stimulation (e.g., Melcher et al. 2012; Jefferies et al. 2008; Kuhbandner and Zehetleitner 2011; Van Steenbergen et al. 2010). An affective state can be described as existing in two-dimensional, orthogonal space, in which valence and arousal constitute each (core) affective state (c.f., Brunyé et al. 2009; Russell 2003). Valence represents unpleasant vs. pleasant feelings towards our state at a particular moment. Arousal describes an energetic side of an affective state at a particular time and is sometimes referred to as intensity or energy level. This energy expresses the degree of excitement or activation an individual feels toward a given stimulus (Montefinese et al. 2014). thus, arousal level could be treated as the property of a stimulus that influences the current affective state, which varies from calm to completely excited (Russell 2003). Factors of valence and arousal were found in the semantic differential approach (Osgood et al. 1957) to understanding emotional reactions to stimuli, as two of the three most important attributes responsible for understanding of affective meaning. Kuhbandner and Zehetleitner (2011) found that both dimensions produce an independent effect on executive control; thus, it is a good strategy to investigate them separately to avoid situations when a single, more pronounced dimension will overwhelm the effects of the less salient one. Valence was found to modulate interference control both in flanker tasks (Melcher et al. 2012; Van Steenbergen et al. 2010) and emotional Stroop tasks (c.f., Nigg 2000; McKenna and Sharma 2004). however, at the same time, arousal was found to modulate processing in those tasks (e.g., Larsen et al. 2006). Russell (2003) stated that core affect was influenced by each object that was charged by valence and arousal; thus, simply watching the affectively charged materials should change the current affective state for a moment (Bojarska 2013). According to Yerkes-Dodson’s law (1908). it is likely that energy load (arousal) should influence interference control. This law describes the relationship between energetic aspects of functioning described as motivation, arousal or excitement, and performance. The energy (arousal) should interplay with performance in a quadratic fashion. This means that, both for low and high energy load states, performance is poor, while, in the case of optimal or medium energy levels, we may observe peaking performance. Because of this, it is important to provide at least three levels of arousal to test the quadratic relationship.

### Aim and Hypothesis

Arousal was found to be the crucial dimension engaged in many psychological processes: from attention control in a rapid visual sequence (Jefferies et al. 2008) to negotiation (Brown and Curhan 2013). Also, executive control was found to be sensitive to arousal level (Kuhbandner and Zehetleitner 2011). working independently of valence. The aim of this paper is to answer the question of whether merely reading verbal material differing in arousal quality could influence the ability to control the interference of features of visual objects presented close to the target object. To measure interference, I decided to use flanker stimuli perceptual competition in a flanker task (Eriksen and Eriksen 1974). Although previous studies using two levels of arousal (Van Steenbergen et al. 2010) failed to report arousal effects on flanker task performance, I expected that arousal would modulate participants’ ability to control visual competition in a way that follows Yerkes-Dodson’s law (1908). I expected that both low and high arousal words would lead to less effective performances in comparison to medium arousal words; thus, one had to choose at least three levels of arousal to search for its consequences for behavior. Otherwise, it would be possible to discover negative results just because chosen arousal levels were equally distanced from the optimal level in a reversed U-shaped function, as described by Yerkes and Dodson (1908).

## Method

### Participants

Sixty participants (30 females and 30 males) aged from 18 to 26 (*M* = 21.46, *SD* = 1.93) were invited to join the study and participated voluntarily in exchange for small gifts. The sample size was determined in advance as 60 participants. All were native Polish language speakers and had normal, or corrected to normal vision. They were students at different Warsaw universities and colleges in equal proportions of humanities, social, life, and natural and engineering sciences. Data from 11 individuals were excluded from further analysis; six individuals were excluded due to their performance rate of about 0.5 accuracy and another five due to non-compliance with the instructions, or technical problems with recorded data files. Finally, data from 49 participants (24 females and 25 males) aged from 18 to 26 (*M* = 21.59, *SD* = 2.01) were analyzed.

## Materials

### Emotional Quality of Words

A list of 135 Polish nouns (3 × 45 words) with known affective qualities was chosen to develop a manipulation allowing to contrast three distinct arousal levels. Words were chosen from among 4905 words introduced by Affective Norms for Polish Words Reloaded (Imbir, submitted), the new normative study for large sets of verbal stimuli. The affective norms of words on this list were determined using a methodology similar to that used in a previous pilot study of affective norms for 1586 words (Imbir 2015). Correlations in ratings for smaller and bigger lists for words included in both were huge and significant (valence *r* = 0.97, arousal *r* = 0.8 and subjective significance *r* = 0.77); thus, these new norms are reliable. For each dimension measured in ANPR_R (Imbir submitted), participants in the normative study (a different group from the sample invited for the current study) assessed their first impression of each word with respect to a number of affective dimensions using a nine-point Likert Self-Assessment Manikin (SAM; Lang 1980). These data were used to select words in three arousal load categories: (1) words that induced a small arousal level, *M* = 3.22, *SD* = 0.24; words that induced a moderate arousal level, *M* = 3.85, *SD* = 0.28; and words that induced a high arousal level, *M* = 4.87, *SD* = 0.41. This approach allows to choose three small intervals from a whole continuous scale of arousal intensity. Those intervals were distinct from one another and separated by unchosen levels of arousal, thus providing an opportunity to inspect consequences of three points on cognitive control. In selecting the words, I controlled for other factors that might influence the scope of attention and flanker task performance, such as valence, concreteness, subjective significance and lexical properties such as frequency in Polish (Kazojć 2011) or number of letters (word length). The levels of those dimensions were moderate and chosen to be equal for each of three arousal categories. The full list of words used, together with their English translations as well as their affective ratings, are included in Appendix.

A one-way ANOVA with arousal (three levels) as the between-subjects factor was used to check that the words chosen for the manipulation were appropriate. There was a main effect of arousal on arousal ratings: *F*(2132) = 305.53, *p* < 0.001, *η* ^*2*^ = 0.82. A simple contrast analysis confirmed the differences between the groups of weakly and moderately arousing words (*F*(1,88) = 128.48, *p* < 0.001, *η* ^*2*^ = 0.59) and between moderately and highly arousing words (*F*(1,88) = 190.22, *p* < 0.001, *η* ^*2*^ = 0.68). The huge *η* ^*2*^ values suggest that levels chosen were, in fact, distinct from one another.

Similar analyses of the effects of the controlled factors revealed no main effect of arousal level group on valence (*F*(2132) = 1.47, *p* = 0.23, *η* ^*2*^ = 0.022; *M* = 5.26, *SD* = 0.61); concreteness (*F*(2132) = 0.027, *p* = 0.97, *η* ^*2*^ = 0.0001; *M* = 4.08, *SD* = 0.88); or subjective significance (*F*(2132) = 0.61, *p* = 0.55, *η* ^*2*^ = 0.009; *M* = 3.76, *SD* = 0.82) ratings. Word frequency data (Kazojć 2011) were natural log-transformed (c.f., Heathcote et al. 1991) as the data were right-skewed (words occurring only once in a wide range of Polish texts were over-represented in the data set). There was no main effect of arousal load groups in the case of word frequency (*F*(2132) = 1.24, *p* = 0.29, *η* ^*2*^ = 0.02; *M* = 6.38, *SD* = 1.67). There was also no main effect of arousal load in the case of word length (*F*(2132) = 0.58, *p* = 0.56, *η* ^*2*^ = 0.009; *M* = 6.36 letters, *SD* = 1.96).

These analyses confirmed that the manipulation words were sufficiently contrasted by three distinct categories of arousal load and were well matched, as they did not differ systematically with respect to other factors that might influence scope processing in flanker interference tasks such as frequency of appearance, word length, valence, concreteness and subjective significance. Any category differences could confidently be attributed only to the intended arousal manipulation.

### Flanker Task

The competition of accompanying object features in vision is the scope of interest of cognitive psychology focusing on understanding visual scene perception. The simplest measure of this phenomenon is provided by the classical flanker task (Eriksen and Eriksen 1974). introduced to measure a slowing of responses to central, target stimuli caused by flanker congruent and incongruent accompanying stimuli. In the current study, a computerized version of the flanker task was used, involving the detection of the appearance of the letter N whilst ignoring the letter H. The target letter (N or H) was displayed in the middle of the screen. The task was to answer the question: ‘Is the central letter N?’ by pressing one of the keys labeled ‘Yes’ and ‘No’. The target letter was flanked by four other letters (two on the right and two on the left), which were the same as the central letter in the congruent trials, and different in the incongruent trials. Both letters N and H share several similar perceptual features (c.f., Eriksen and Eriksen 1974). thus, this version of the task is most sensitive to visual competition. Visual competition concerns the spatial distance of the flanker letter; for that reason, three levels of difficulty were applied. The most difficult conditions are those when the flanker letters are close to each other and the target letter, while the easiest conditions are when the target letters are distant. In the version of the test used, a high difficulty of the task was designated by placing flanker letters a distance of 4 % and 8 % of the screen length from the middle of the screen position (50 %) on the left and right. In the medium difficulty version, flankers were placed +/− 6 % and +/−12 % of the screen length, while, in the easiest conditions, they were placed +/− 8 % and +/−16 % of the screen length from the middle. Those parameters were chosen based on pilot study data.

The flanker task itself also provides two types of trials. In congruent trials, flanker letters are the same as the target letter (e.g., N flanked by N’s, or H flanked by H’s). In incongruent trials, the flanker letter is different than the target (e.g., N flanked by H’s, or H flanked by N’s). These types of trial allow us to measure cognitive control effects upon performance (c.f., Van Steenbergen et al. 2010). Incongruent trials are harder for the participants because of visual interference; for that reason, reaction times are longer and response accuracy is smaller in incongruent trials (Eriksen and Eriksen 1974). By subtracting accuracy or reaction times for congruent from incongruent trials, we can obtain conflict effect measures (Van Steenbergen et al. 2010). Finally, the flanker task provides ‘Yes’ and ‘No’ expected answer trials with, respectively, N or H presented in the target position. Typically a ‘Yes’ answer is preferable for participants (c.f., Yes-bias: a tendency to saying yes for all appearing questions asked in psychological surveys, even when the probability of both answers is equal); thus ‘No’ key pressing was expected to require additional cognitive control.

### Apparatus

A standard 17-in. laptop computer was used. The experimental protocol was designed using E-Prime 2.0 software. Participants answered using the computer keyboard with the keys designed to be active during the session marked by stickers.

### Design and Procedure

**Fig. 1:**
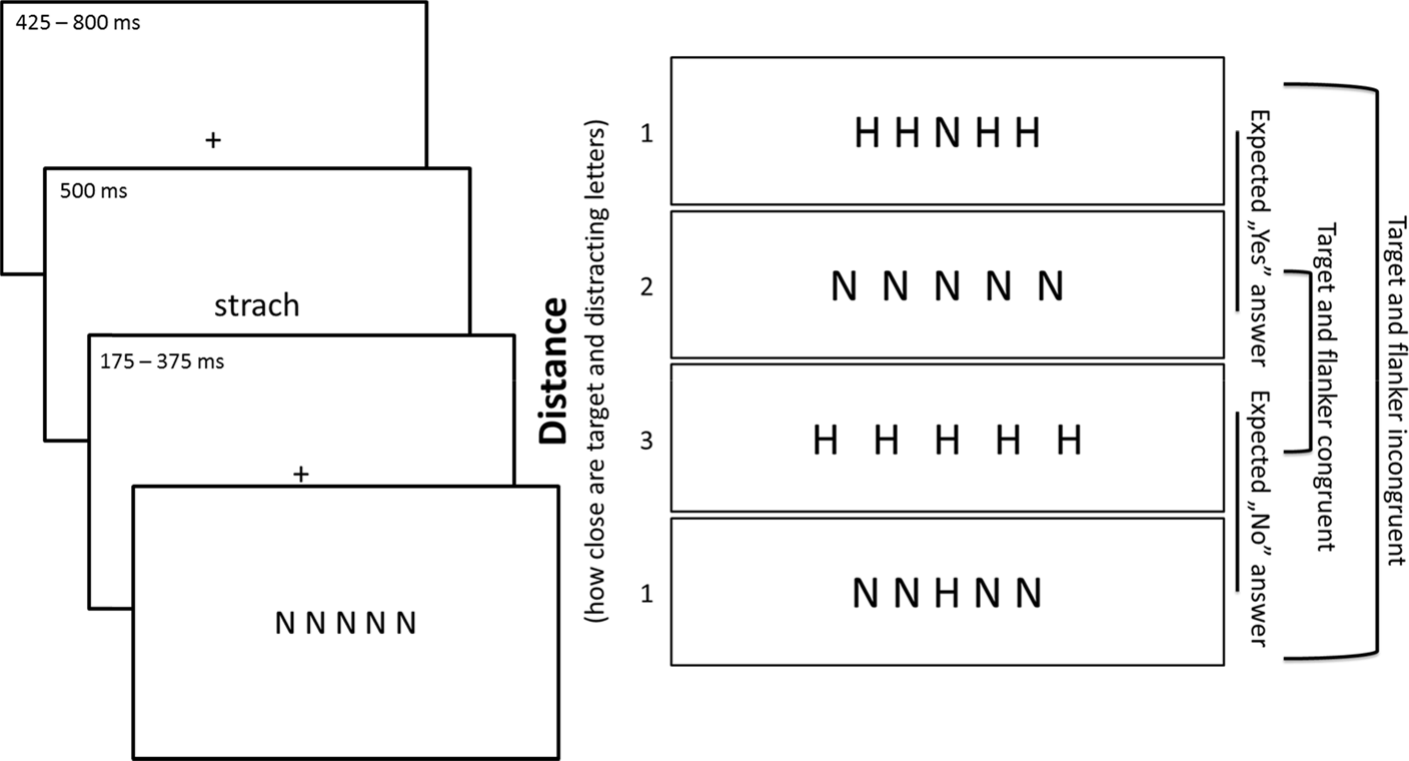
Single trial of experimental procedure and types of flanker task trials, including task difficulty(3 levels), target and flanker congruency type (2 levels) and expected correct answer (2 levels)

The study was designed as a 3 (arousal of words) × 3 (task difficulty) × 2 (type flanker for the trial: congruent or incongruent) × 2 (type of expected correct answer: Yes or No) within-subject experimental design. All participants read all 135 words assigned to the three levels of arousal groups in a random order, and then, after each word, performed 135 trials of the flanker task, also presented in a random order (with N or H as the central letter, plus congruent or incongruent flankers). Each word from the list in Appendix appeared only once during the experiment; thus, in single cells of experimental schema, there was a mean expected value of 3.75 trials (words). Figure 1 presents single trial and types of flanker task variants.

The first step of the procedure comprised a simple perceptual task concerning the detection of a red dot appearing to the right or left of the fixation point. Each participant performed 135 trials of this task, which enabled the alignment of the participants’ initial affective states. The practice session consisted of a single trial of the flanker task, designed to provide training for the task. Participants were instructed that, during the experiment, some words would appear for a brief moment (500 ms). There was no special instruction concerning the words, only simply to read them. The single experimental trial consisted of (1) a fixation cross displayed in the middle of the screen for a random time lasting from 425 to 800 ms (with increments of 25 ms). Then (2), a word was displayed for 500 ms, which was then replaced by (3) another fixation cross lasting a random time, from 175 to 375 ms (with increments of 25 ms). Finally, (4) the flanker task appeared (as described above). This task lasted until the participant responded, when it was replaced by the fixation point of the next trial. After all 135 trials of flanker task procedures had been completed, participants performed another unrelated task and completed questionnaires (not discussed here and that did not affect the results of this part of the study).

### Data Treatment and Analytic Strategy

Data were gathered from a total of 6615 trials involving the 49 participants. Outliers were excluded (RT > 3*SD* (2180 ms) or RT < 250 ms, *N* = 58, no more than 0.8 % of all trials). The overall error rate was 3.7 %; the mean reaction time was 616 ms (*SD* = 238 ms). To test the hypotheses concerning flanker interference in the flanker task, I conducted a repeated measures ANOVA involving the following: 3 (arousal of words) × 3 (task difficulty) × 2 (type flanker for the trial: congruent or incongruent) × 2 (type of expected correct answer: Yes or No). First, I analyzed the response accuracy, then reaction times, and finally, using the interference effect index (see below), I conducted analyses concerning reaction times using logarithm natural transformation (*ln*) of reaction times. Each trial reaction time was transformed by *ln*, then data across conditions were aggregated. Logarithm natural transformation is a standard procedure for reaction time data allowing the analysis of right-skewed (c.f., Heathcote et al. 1991) distribution and using parametric statistics. The results, summarized in the Figures, are presented in raw form (ms).

To measure the cognitive conflict elicited by the congruent and incongruent trials, the interference effect index was assessed (c.f., Van Steenbergen et al. 2010) for response accuracy and reaction times using a formula in which the first symbol means target letter, while the second is a flanker: ((NH-NN) - (HN-HH)). In other words, in the ‘Yes’ and ‘No’ trials, I subtracted the scores of the congruent from those of the incongruent trials, then subtracted the results of the ‘No’ from the ‘Yes’ conditions. The idea was to provide change measures by subtracting scores of the easiest from the hardest trials (c.f., Van Steenbergen et al. 2010). Using this index, an additional repeated measures ANOVA was conducted involving the following: 3 (arousal of words) × 3 (task difficulty) factors.

## Results

### Response Accuracy

Taking into account response accuracy, I found no statistically significant main effect of arousal level: *F*(2,47) = 0.85, *p* = 0.43, *η* ^2^ = 0.035, but a significant main effect of flanker distance: *F*(2,47) = 6.14, *p* = 0.004, *η* ^2^ = 0.21. In the case of close distracters presentation, the mean response accuracy was *M* = 0.95 (*SEM* = 0.006); for medium distracters presentation, the accuracy was *M* = 0.97 (*SEM* = 0.004); and for distant presentation, *M* = 0.97 (*SEM* = 0.004). The polynomial contrast showed a linear relationship: *F*(1,48) = 10.52, *p* = 0.002, *η* ^2^ = 0.18. The simple contrast analysis showed that the difference in response accuracy was significant between close and medium presentations of distracters (*F*(1,48) = 10.46, *p* = 0.002, *η* ^2^ = 0.18) and between close and distant presentations of distracters (*F*(1,48) = 10.52, *p* = 0.002, *η* ^2^ = 0.1). I found a statistically significant main effect for type of answer expected: *F*(1,48) = 7.42, *p* = 0.009, *η* ^2^ = 0.134. Expected ‘Yes’ trials (target letter N) were less accurate: *M* = 0.96 (*SEM* = 0.007) than expected ‘No’ trials with H as the target letter: *M* = 0.98 (*SEM* = 0.003). I found a statistically significant main effect of trial congruency: *F*(1,48) = 6.78, *p* = 0.012, *η* ^2^ = 0.124. Congruent trials were performed more accurately: *M* = 0.96 (*SEM* = 0.005) in comparison to incongruent trials: *M* = 0.97 (*SEM* = 0.004). I found no significant interaction between the factors analyzed.

### Natural Logarithm of Reaction Times

**Fig. 2:**
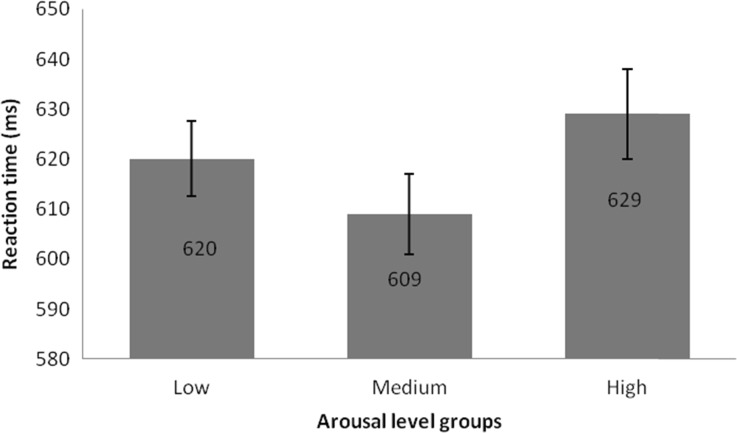
Reaction times (ms) for groups of words differing according to arousal load. Error bars represent SEM

Regarding the *ln* of reaction times, I found a statistically significant main effect of arousal level: *F*(2,47) = 4.56, *p* = 0.015, *η* ^2^ = 0.14. In the case of low arousal words, the response time was *M* = 620 ms (*SEM* = 15 ms); for medium arousal words *M* = 609 ms (*SEM* = 16 ms); and in high arousal words *M* = 629 ms (*SEM* = 18 ms). Polynomial contrast showed a quadratic relationship: *F*(1,48) = 8.41, *p* = 0.006, *η* ^2^ = 0.15. Difference contrast analysis showed slightly significant differences between low and medium arousal words (*F*(1,48) = 3.47, *p* = 0.061, *η* ^2^ = 0.071) and significant differences for the medium and high arousal word groups (*F*(1,48) = 5.16, *p* = 0.028, *η* ^2^ = 0.15). I found no statistically significant main effect of distracters’ distance: *F*(2,47) = 2.09, *p* = 0.135, *η* ^2^ = 0.082. I found a statistically significant main effect for type of answer expected: *F*(1,48) = 17.14, *p* = 0.001, *η* ^2^ = 0.263. Expected ‘Yes’ trials took participants less time (*M* = 607 ms (*SEM* = 16 ms)) than expected ‘No’ trials: *M* = 631 ms (*SEM* = 16 ms). I found a statistically significant main effect of trial congruency: *F*(1,48) = 26.82, *p* = 0.001, *η* ^2^ = 0.36. Congruent trials took participants less time to answer (*M* = 608 ms (*SEM* = 16 ms)) in comparison with incongruent trials: *M* = 630 ms (*SEM* = 17 ms). No significant interaction between factors analyzed was found; thus, I do not present them here. Figure 2 presents the pattern of results for the different levels of arousal groups.

### Interference Index

**Fig. 3:**
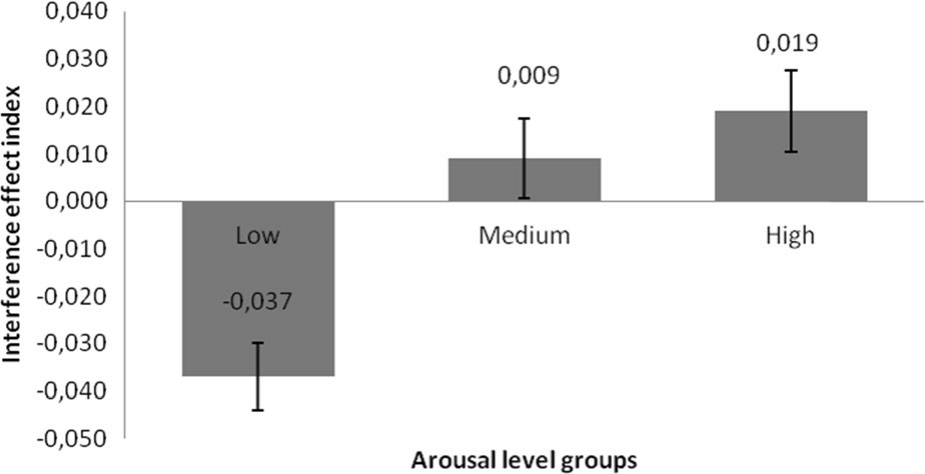
Interference effect index calculated for response accuracy for groups of words differing according to arousal load. Lowest and highest values = major interference; 0 = no interference. Error bars represent SEM

Taking into account the interference effect index for the accuracy of answers, I conducted a 3 (arousal of words) × 3 (task difficulty) repeated measures ANOVA. The type of expected answer (‘yes’ vs ‘no’) and congruency type of trial (congruent vs incongruent) were included in the interference index formula (c.f., Method section). I found a statistically significant main effect of arousal level: *F*(2,47) = 3.7, *p* = 0.032, *η* ^2^ = 0.136. In the case of low arousal words, the index ratio was *M* = −0.037 ms (*SEM* = 0.014); for medium arousal words, *M* = 0.009 (*SEM* = 0.017); and in high arousal, *M* = 0.019 (*SEM* = 0.017). Polynomial contrast showed a linear relationship: *F*(1,48) = 6.71, *p* = 0.013, *η* ^2^ = 0.123. Difference contrast analysis showed significant differences between low and medium arousal words (*F*(1,48) = 4.9, *p* = 0.032, *η* ^2^ = 0.093) and a weakly significant difference between medium and high arousal words (*F*(1,48) = 3.36, *p* = 0.073, *η* ^2^ = 0.065). I found no statistically significant main effect of distracters’ distance (*F*(2,47) = 0.72, *p* = 0.5, *η* ^2^ = 0.03). I found no interaction between arousal and distance factors, and no significant effects were found in the case of the interference effect index calculated for reaction times. Figure 3 presents the pattern of results obtained for the different levels of arousal groups.

## Discussion

The current study demonstrated that reading of words differing in arousal level could influence the processing speed, as well as the interference effect index derived from the response accuracy of the flanker task engaging visual competition. I expected that the arousal level impact on interference control would follow Yerkes-Dodson’s law (1908). According to this law, both low and high levels of activation disturb performance, while medium and optimal levels enhance it. The data obtained were consistent with Yerkes-Dodson’s law. In fact, stimuli of medium arousal load presented to participants resulted in the shortest reaction times and close to a zero interference effect index for response accuracy. Both low and high arousal words were associated with a lengthening of reaction times and a distancing from a neutral interference effect index. Response latencies in this task represented the difficulty for interference inhibition of competing letter features. This claim was supported by the main effect of congruency of target and flanker stimuli. Incongruent trials of higher interference were processed longer than the congruent trials. This result corresponded with that of Chajut et al. (2009). In this sense, low and high arousal disturbed interference, probably making it hard to maintain in high-arousal conditions, or hard to achieve in low arousal conditions.

Although there was no arousal effect on response accuracy, it appeared that, by using the interference effect index (c.f., Van Steenbergen et al. 2010) on this data, we could see some interesting relationships. The construction of an interference effect index provided us with a measure of relative interference (see Data Treatment and Analytic Strategy). It was rather obvious that incongruent trials stimulated more interference than congruent ones. Subtracting one from the other, we found more differences attributed to the flanker stimuli competition. Furthermore, interpreting the higher error rates and quicker answers given, I found that ‘Yes’ answers were more expected by participants than ‘No’ answers (simply ‘Yes’ answer was dominant while ‘No’ required additional cognitive control to appear, which could be related to so-called ‘Yes bias’). By subtracting mere interference in the ‘No’ from the ‘Yes’ conditions, we could identify the conditions in which interference was stronger. If the index value was negative, trials with the target H letter requiring a ‘No’ answer produced higher interference. In the opposite situation, when the index value was positive, trials with the target N letter requiring a ‘No’ answer produced higher interference. As can be seen in Fig. 3, low arousal words strengthened interference in the ‘No’ trials, whereas high arousal words strengthened interference in the ‘Yes’ trials. Both were distant from point zero, indicating no interference, as can be seen in association with the medium arousal words. This pattern of results was partly represented in the study by Van Steenbergen et al. (2010). The experimental schema used by the authors represented only two levels of arousal; they found that the highest index value was associated with accuracy in the group of participants showing anxiety (low pleasure and high arousal), and the lowest in the group with elicited sadness (low pleasure and low arousal). As in the present study, this relationship was not observed in the reaction time data. The different effects for low and high arousal stimuli could be interpreted in terms of motivational processes (c.f., Gable and Harmon-Jones 2010). Low arousal stimuli could lower motivation intensity, therefore making it desirable for compliance with the task ‘Yes’ answer less salient, thus reducing interference in this category of trials.

I believe that the current findings could shed new light on the activation mechanisms underlying cognitive and interference control. As a recent study using the same methodology has shown, the effect is not limited to cognitive control, but also shapes global vs. local cognitive scope preference (c.f., Brunyé et al. 2009). Altogether, the findings presented in this article suggest that the arousal level of words read influences immediately following spatial interference control. The material used to manipulate arousal level was carefully chosen and controlled for other important factors such as valence, subjective significance, concreteness, frequency and length. The novelty of results presented was that the quadratic relationship was found to be crucial for the understanding of arousal/energy consequences for cognition. Although this expectation was introduced by Yerkes and Dodson (1908). modern studies concerning consequences of arousal mostly focus on only two instead of three levels of arousal (e.g., Kuhbandner and Zehetleitner 2011; Van Steenbergen et al. 2010). thus, research sometimes does not discover all arousal effects. From a theoretical point of view, it was possible to demonstrate no arousal effects when two chosen points of intensity were similar in distance from the optimal level of arousal. Presented results suggested that the quadratic function best represented the relationship between an energetic aspect of mind and performance in the cognitive domain, and at least three levels of arousal should be included in experimental schema in order to not miss it.

## Appendix

Table present list of all words (in Polish version and translated into English) used to create factorial manipulation (3 arousal levels) as well as ratings from ANPW_R - normative study for 4905 Polish words concerning arousal, subjective significance, valence, concreteness and lexical variables such as frequency of appearance based on Kazojć (2011) dataset and length (number of letters)

**Table:**

Polish word	English translation	arousal category	Arousal M	Significance M	Valence M	Concreteness M	Frequency of appearance	Number of Letters
arka	ark	1	3.08	3.32	5.74	3.08	138	4
aspekt	aspect	1	2.84	3.48	5.30	6.18	555	6
czyn	deed	1	3.44	5.04	5.66	4.96	1709	4
dokument	document	1	3.22	4.48	5.30	3.00	1863	8
echo	echo	1	3.42	3.14	5.64	4.08	2696	4
emerytura	pension	1	3.42	4.36	4.90	4.18	45	9
fundusz	fund	1	3.18	3.60	5.72	4.22	308	7
gamma	gamma	1	3.06	3.30	5.12	4.73	232	5
gleba	soil	1	3.18	3.34	4.94	2.36	228	5
godzina	hour	1	3.14	4.50	5.16	5.10	4687	7
istota	being	1	3.26	5.02	5.50	4.76	4054	6
jednostka	unit	1	3.00	4.60	5.02	4.08	919	9
kawałek	chunk	1	2.94	3.52	5.16	3.65	8697	7
klan	clan	1	3.24	2.70	5.26	3.60	539	4
kolor	color	1	3.40	3.70	6.04	4.88	3616	5
krok	step	1	2.96	4.30	5.68	3.14	12,259	4
laik	layman	1	3.24	3.50	4.43	5.02	52	4
lekcja	lesson	1	3.24	4.70	4.98	3.78	575	6
mgiełka	haze	1	2.88	3.28	5.88	2.82	494	7
mieszkaniec	inhabitant	1	3.18	3.46	5.38	2.78	602	11
mila	mile	1	2.86	2.66	5.46	3.96	781	4
milczenie	silence	1	3.26	4.31	4.60	4.92	5840	9
namiot	tent	1	3.56	2.92	6.24	2.04	1206	6
orbitowanie	orbit	1	3.90	2.70	5.22	4.72	2	11
osoba	person	1	3.72	5.16	5.82	3.72	5460	5
pasmo	band	1	3.14	3.02	5.38	3.54	1104	5
pauza	pause	1	3.08	3.12	5.04	4.92	552	5
poezja	poetry	1	2.78	4.48	6.06	5.08	955	6
połysk	shine	1	3.28	3.20	5.80	3.90	407	6
północ	north	1	3.37	4.16	5.76	4.68	6813	6
prasowanie	ironing	1	3.20	3.04	4.84	3.36	23	10
rzemiosło	craft	1	3.36	3.58	5.60	4.50	336	9
saga	saga	1	3.12	3.32	5.56	4.20	770	4
seria	series	1	3.26	2.84	5.28	4.70	1180	5
sfinks	sphinx	1	3.00	2.42	5.62	3.26	176	6
singiel	single	1	3.84	4.10	4.88	3.74	6	7
smuga	streak	1	3.32	3.12	4.82	3.30	931	5
tenor	tenor	1	3.32	2.18	5.44	3.60	353	5
trasa	route	1	3.42	3.84	5.50	3.04	477	5
wersja	version	1	2.86	2.58	5.22	5.38	965	6
woń	odor	1	3.26	4.20	5.44	3.76	2810	3
wykonywanie	implementing	1	3.28	4.40	5.26	4.96	243	11
zasada	rule	1	3.16	4.58	5.40	5.38	1241	6
zdanie	sentence/sense	1	3.30	4.40	5.70	4.52	7177	6
zero	zero	1	3.00	3.12	4.02	4.42	1693	4
akcent	accent	2	3.58	3.06	5.58	4.66	1076	6
Biblia	Bible	2	3.52	5.00	5.68	3.00	378	6
budżet	budget	2	4.30	4.34	5.32	3.48	243	6
bufor	buffer	2	3.47	2.78	5.20	3.88	42	5
chrzest	baptism	2	3.74	3.78	5.72	4.90	309	7
dystans	distance	2	3.58	4.44	4.84	4.68	1461	7
firma	business	2	3.82	4.32	5.66	3.06	1505	5
głębia	profundity	2	3.74	4.36	5.26	5.48	243	6
gromada	troop	2	3.90	2.78	5.40	3.36	1175	7
grupa	group	2	3.90	3.90	5.84	3.32	5460	5
hrabia	count	2	3.58	2.92	5.32	3.28	5345	6
imam	imam	2	3.59	2.85	5.00	4.14	130	4
interes	business	2	4.36	3.92	5.86	4.82	3421	7
jazda	ride	2	4.20	3.62	5.82	3.44	2101	5
lekarstwo	medicine	2	3.52	4.98	5.40	2.88	1126	9
loteria	lottery	2	4.10	3.12	5.76	3.46	56	7
marszałek	marshal	2	4.32	3.20	5.10	2.84	1315	9
mrugnięcie	wink	2	3.46	3.60	5.78	3.56	92	10
nawyk	habit	2	3.74	4.46	4.88	5.64	367	5
obserwowanie	observation	2	3.56	4.28	5.42	4.84	219	12
odcień	tint	2	3.48	3.14	5.52	4.80	1024	6
olbrzym	giant	2	4.04	2.70	4.98	3.24	1495	7
plemię	tribe	2	3.92	3.14	5.32	3.66	1018	6
poganin	heathen	2	3.66	2.94	4.64	5.10	95	7
pokaz	show	2	3.88	3.70	5.74	4.12	1035	5
posag	dowry	2	3.68	3.26	5.58	2.71	332	5
posiadacz	possessor	2	3.88	3.64	5.82	4.28	212	9
powieść	novel	2	3.50	3.96	6.08	3.88	2552	7
praca	labor	2	4.12	5.74	5.90	4.10	6395	5
profesor	profesor	2	4.14	4.74	5.86	3.16	8262	8
próba	attempt	2	3.76	4.12	5.30	4.90	2756	5
sabat	Sabbath	2	4.10	2.71	4.56	5.12	913	5
sługa	servant	2	3.72	3.20	4.32	3.58	1668	5
swada	zest	2	3.68	3.51	4.77	5.00	19	5
szczegół	detail	2	3.50	4.48	5.44	4.74	1358	8
szlachta	nobility	2	4.08	2.78	5.46	3.90	811	8
ulewa	downpour	2	4.00	3.46	4.18	2.74	495	5
uwaga	note	2	4.42	5.02	4.88	5.60	3261	5
waga	weight	2	3.70	3.80	4.72	3.06	429	4
wpływ	influence	2	3.80	4.47	5.28	5.32	3586	5
wygląd	appearance	2	4.32	4.66	5.82	4.58	4185	6
wyłom	breach	2	4.02	3.06	4.42	3.20	278	5
wymiana	exchange	2	3.68	4.30	5.45	4.46	792	7
zadatki	smack	2	3.80	3.68	5.32	5.34	94	7
zaułek	alley	2	4.28	3.64	4.24	2.98	444	6
alarm	alert/alarm	3	4.48	4.80	4.16	3.42	1646	5
bieganie	running	3	4.66	4.04	5.94	3.24	90	8
buntownik	rebel	3	5.54	4.86	4.80	4.24	101	9
burza	storm	3	5.30	4.48	4.86	3.06	3238	5
car	tsar	3	4.56	2.36	4.82	2.64	1073	3
doping	doping	3	5.18	3.30	5.24	4.46	23	6
duch	spirit	3	4.76	3.24	4.16	5.46	5226	4
dziewica	virgin	3	5.00	4.18	6.18	3.38	446	8
ekstrawertyk	extrovert	3	5.17	4.59	5.28	5.64	6	12
galop	gallop	3	4.56	2.54	5.76	3.62	252	5
geniusz	genius	3	4.76	5.42	7.22	5.50	936	7
harem	harem	3	4.74	2.86	5.14	3.75	111	5
hazard	gamble	3	5.30	3.16	3.96	4.04	292	6
karykatura	pamphlet/caricature	3	4.20	2.50	5.56	3.88	131	10
koszary	barracks	3	4.72	2.82	4.44	2.96	384	7
kryminał	thriller/jail	3	5.06	3.28	4.88	3.54	111	8
labirynt	maze	3	4.52	3.68	5.16	3.20	1031	8
lesbijka	lesbian	3	5.06	3.88	4.76	3.96	26	8
majątek	fortune	3	5.04	5.28	6.54	4.02	2861	7
maniak	maniac	3	4.92	4.34	4.30	5.20	212	6
młodzież	youth	3	4.66	3.98	5.68	3.40	1703	8
mrok	gloom	3	4.48	4.06	4.18	4.24	3909	4
mutacja	mutation	3	4.64	3.30	4.42	4.34	100	7
ogień	fire	3	4.80	5.04	5.46	2.80	11,105	5
orgia	orgy	3	5.82	3.48	4.90	4.30	108	5
parada	parade	3	4.08	2.76	5.64	3.42	190	6
płomień	flame	3	4.90	4.30	5.92	3.08	2856	7
poprawka	amendment	3	4.52	4.46	3.90	4.16	121	8
promieniowanie	radiation	3	4.66	3.76	4.24	4.32	705	14
salwa	salvo	3	4.70	2.74	5.56	3.51	332	5
sesja	session	3	5.06	4.54	3.76	4.34	253	5
smok	dragon	3	4.58	2.80	5.66	4.18	3438	4
sprint	sprint	3	4.54	3.28	5.68	3.64	38	6
strzał	shot	3	5.04	3.94	4.56	3.14	3675	6
szaleństwo	craze	3	6.28	4.48	5.66	6.48	2654	10
turbo	turbo	3	5.06	3.78	5.76	5.33	30	5
wampir	vampire	3	4.90	3.00	4.52	4.42	701	6
władza	authority	3	4.76	5.04	5.18	5.80	2056	6
wojsko	army	3	4.96	4.16	4.94	2.90	2893	6
wolt	volt	3	4.31	2.41	5.08	4.44	40	4
wynik	result	3	4.60	5.16	5.52	4.58	1919	5
wysiłek	effort	3	5.14	5.10	5.26	4.52	2024	7
wyścig	race	3	4.70	3.94	5.28	3.58	455	6
zombie	zombie	3	4.80	2.36	3.94	4.58	185	6
zwycięzca	winner	3	5.56	5.86	7.42	5.24	336	9
